# FAK-Activated Mucosal Healing Promotes Resistance to Reinjury

**DOI:** 10.3390/cells15010016

**Published:** 2025-12-22

**Authors:** Sema Oncel, Guiming Liu, Louis Kwantwi, Emilie E. Vomhof-DeKrey, Ricardo Gallardo-Macias, Vadim J. Gurvich, Marc D. Basson

**Affiliations:** 1 Department of Biomedical Sciences, University of North Dakota School of Medicine & Health Sciences, Grand Forks, ND 58203, USA; tech@oncel-llc.com (S.O.);; 2 Department of Biomedical Sciences, Northeast Ohio Medical University College of Medicine, Rootstown, OH 44272, USA; gliu@neomed.edu (G.L.); lkwantwi@neomed.edu (L.K.); 3 Department of Surgery, University of North Dakota School of Medicine & Health Sciences, Grand Forks, ND 58203, USA; 4 Department of Pathology, University of North Dakota School of Medicine & Health Sciences, Grand Forks, ND 58203, USA; 5 Institute for Therapeutics Discovery and Development and Department of Medicinal Chemistry, College of Pharmacy, University of Minnesota, Minneapolis, MN 55414, USAvadimg@umn.edu (V.J.G.)

**Keywords:** focal adhesion kinase, nonsteroidal anti-inflammatory drug, gastrointestinal injury, angiogenesis

## Abstract

**Highlights:**

**What are the main findings?**

- FAK activation accelerates ischemic ulcer healing, in part by enhancing angiogenesis.
- FAK activation during an initial injury reduces susceptibility to recurrent NSAID-induced intestinal injury.

**What are the implications of the main findings?**

- FAK activation may represent a novel therapeutic avenue for gastrointestinal injury.
- FAK activation may be especially valuable for patients requiring long-term NSAID therapy.

**Abstract:**

Background: Gastrointestinal (GI) mucosal injury is a frequent complication of long-term nonsteroidal anti-inflammatory drug (NSAID) use. Effective mucosal healing requires coordinated epithelial migration, proliferation, and angiogenesis, which may be influenced by focal adhesion kinase (FAK). This study aimed to determine whether our newly developed FAK activators promote intestinal mucosal healing by enhancing angiogenesis and whether FAK activation increases resistance to reinjury. Methods: Ischemic jejunal ulcers were induced in C57BL/6 mice. After 24 h, mice received intraperitoneal injections of the FAK activator ZINC40099027 (ZN27, 900 µg/kg every 6 h) or vehicle for 2, 4, or 14 days. Ulcer areas were quantified, and liver and kidney function were assessed. Ulcer and adjacent tissues were analyzed by immunofluorescence staining for angiogenesis and proliferation markers. In vitro, human umbilical vein endothelial cells (HUVECs) were treated with ZN27 to evaluate proliferation, migration, angiogenesis, and intracellular signaling. In a reinjury model, male C57BL/6J mice received continuous infusion of the FAK activator M64HCl (25 mg/kg/day) or vehicle for 7 days, with a single subcutaneous injection of indomethacin (10 mg/kg) on day 1 to induce GI injury. Fourteen days after the first dose of indomethacin, the mice received a second indomethacin challenge, and one day later, total ulcer areas in the pyloric opening and small intestine were quantified. Results: Ulcer areas were significantly smaller in ZN27-treated mice compared with vehicle-treated controls at 3 and 5 days, accompanied by increased expression of angiogenesis and proliferation markers. In vitro, ZN27 enhanced HUVEC migration via FAK activation in an ERK1/2-dependent manner and increased the number of angiogenic sprouts. In the reinjury model, treatment with M64HCl during the initial indomethacin-induced injury resulted in significantly smaller ulcer areas in both the pyloric opening and small intestine after the second indomethacin challenge compared with controls. Conclusions: FAK activation accelerates ischemic ulcer healing, in part by enhancing angiogenesis. Moreover, FAK activation during an initial injury reduces susceptibility to recurrent NSAID-induced intestinal injury, perhaps because it promotes initial higher-quality ulcer repair.

## 1. Introduction

The gastrointestinal (GI) mucosa may be injured by infections, medications, or chronic conditions such as peptic ulcer disease, Crohn’s disease, and ulcerative colitis. Nonsteroidal anti-inflammatory drugs (NSAIDs) are widely used for their anti-inflammatory and analgesic effects, accounting for approximately 8% of prescriptions globally [1]. In the United States alone, more than 111 million NSAID prescriptions were issued in 2000, with an estimated cost of $4.8 billion [2,3]. Among individuals aged 65 years and older, 70% reported weekly use of NSAIDs and/or aspirin, and 34% reported daily use [4]. NSAID use is associated with a 2- to 6-fold increased risk of gastrointestinal (GI) complications [5]. Notably, up to 70% of patients with long-term NSAID use exhibit endoscopic abnormalities, despite only 10% reporting dyspeptic symptoms [1].

NSAID-related GI complications can affect the upper GI tract (up to the second part of the duodenum) as well as the mid- and lower GI tract [6]. NSAID-induced gastropathy and enteropathy are characterized by mucosal erythema, erosions, ulceration, hemorrhage, perforation, annular constriction ring formation, and, in severe cases, small bowel obstruction [7]. Strategies for prevention and management include using the lowest effective NSAID dose, co-administration of proton pump inhibitors (PPIs), the prostaglandin analog misoprostol, and eradication of *Helicobacter pylori* infection [7].

Historically, clinical efforts focused on preventing upper GI complications. However, increasing attention is being directed toward NSAID-induced enteropathy, especially with the introduction of enteric-coated NSAID formulations. Although the use of gastroprotective agents and declining *Helicobacter pylori* prevalence have reduced the incidence of upper GI complications, the incidence of lower GI complications is rising [8]. Recent studies report an elevated risk of lower GI bleeding among patients using both NSAIDs and PPIs [9,10,11]. In patients undergoing video capsule endoscopy, up to 70% showed small bowel mucosal injury—including erosions, ulceration, mucosal hemorrhage, and inflammation—after NSAID use [12].

NSAIDs disrupt the intestinal barrier by inhibiting cyclooxygenase and damaging mitochondria, facilitating the translocation of enteric bacteria into the mucosa. Gram-negative bacteria and endogenous danger signals then initiate inflammatory cascades that contribute to ulcer formation [7,13,14]. Chronic inflammation impairs wound healing, delaying repair and promoting disorganized tissue remodeling. While misoprostol has demonstrated efficacy in treating NSAID-induced small intestinal ulcers, its overall healing rate is low, and it can cause side effects such as diarrhea and abdominal pain. Therefore, novel treatments should be developed to directly promote mucosal healing and restore the integrity of the mucosa as early as possible.

While elimination of ulcers is the treatment goal, the quality of healing is equally important, as it determines susceptibility to reinjury and recurrence. This concept is well established in clinical settings such as peptic ulcer disease [15], inflammatory bowel disease [16], and their corresponding animal models [17,18], where suboptimal healing is associated with increased rates of relapse and complications. The use of NSAIDs is significantly associated with an increased risk of gastrointestinal (GI) complications in individuals with a history of prior GI events. A meta-analysis reported that the odds ratio (OR) for a first gastroduodenal bleed with NSAID treatment was 2.39 (95% confidence interval, CI, 2.16–2.65), whereas in patients with a prior or unspecified history of GI events, the OR increased to 4.76 (95% CI, 4.05–5.59) [19,20]. These findings suggest that previously healed lesions are more vulnerable to reinjury, especially when healing quality is compromised by delayed recovery, abnormal mucosal structure, excessive fibrosis, or impaired blood supply. Therefore, strategies that promote early and rapid healing resembling the original tissue structure may provide greater resistance to subsequent injury.

Focal adhesion kinase (FAK) is a 125 kDa non-receptor tyrosine kinase, ubiquitously expressed in the gastrointestinal tract, including intestinal epithelial cells, endothelial cells, fibroblasts, and cells within the muscularis layer and localized primarily in the plasma membrane and cytoplasm, and thus pharmacologic FAK activation is expected to influence multiple cellular compartments during mucosal repair. FAK regulates key cellular processes including migration, proliferation, survival, and tumor progression [21,22]. It is predominantly located in focal adhesions—specialized structures that mediate cell-extracellular matrix (ECM) interactions and transduce mechanical and chemical signals. FAK activation facilitates epithelial sheet migration [23]. However, diminished FAK activation has been observed in migrating intestinal epithelial cells in vitro and at the margins of human mucosal ulcers [24], suggesting that enhancing FAK activity may promote mucosal repair. The recent development of small-molecule FAK activators has enabled the investigation of their therapeutic potential in mucosal healing [25,26,27,28,29]. Indeed, FAK activators can promote GI mucosal healing through promoting epithelial migration [25,26,27,28,29].

Gastrointestinal mucosal healing is a tightly regulated process that involves multiple biological events. The initial step is epithelial restitution, during which epithelial cells migrate to cover the denuded area, followed by cellular proliferation to replace lost cells. Angiogenesis is also critical for restoring tissue structure and function [30]. In addition to promoting epithelial cell migration, FAK may influence angiogenesis as well [31,32,33,34], although such studies have typically involved inhibition or silencing of FAK or genetic overexpression as the effects of a specific pharmacologic FAK activator have not been previously investigated. Overall, mucosal healing is orchestrated by complex interactions among signaling pathways and external stimuli [35,36]. Activation of FAK may contribute to one or more of these mechanisms, thereby accelerating ulcer repair. In this study, we investigated whether our newly developed FAK activators promote intestinal mucosal healing by enhancing angiogenesis. Furthermore, we examined whether mice treated with an FAK activator for NSAID-induced injury would exhibit greater resistance to a subsequent NSAID challenge compared to mice that underwent natural healing.

## 2. Materials and Methods

### 2.1. Reagents

Pierce bicinchoninic acid (BCA) Protein Assay Kit (#23225), 0.05% Trypsin-EDTA (#25-300), Medium 199 (10×) (#11825-015), and Medium 199 (1×) (#11150-059), DPBS (10×), calcium, magnesium, (#14080055), DAPI (#D1306) were obtained from Thermo Fisher Scientific (Waltham, MA, USA). IRDye conjugated secondary antibodies were the anti-rabbit IRDye 680 (#925-68073) and anti-mouse IRDye 800 (#925-32213) from LI-COR Biosciences (Lincoln, NE, USA). FAK-Tyr-397 antibody (#ab81298) was from Abcam (San Francisco, CA, USA). Antibodies to total FAK (Anti-FAK, clone 4.47, #05-537) from EMD Millipore (Temecula, CA, USA) and Erk1/2 T-202/Y-204 (#4370), ERK1/2 (#4696), and MEK1/2 inhibitor U0126 (#9903) were from Cell Signaling (Danvers, MA, USA). Collagen type-I (#C8919), 5N NaOH (#S8045), Hydroxyurea (#H8627), Anti-phospho-histone H3 (#06-570), Anti-actin α-smooth muscle-FITC antibody (#F3777) were obtained from Sigma-Aldrich (Verona, WI, USA). The cell counting kit-8 (CCK-8) (#CK04-01) was purchased from Dojindo Molecular Technology (Rockville, MD, USA). Mouse ALT ELISA kit (#MBS264717) was from MyBioSource (San Diego, CA, USA). Mouse creatinine kit (#80350) was from Crystal Chem (Elk Grove Village, IL, USA). Glacial acetic acid (#A38S-500) was from Fisher Chemical (Fair Lawn Industrial Park, NJ, USA). Rat tail collagen type I (#50204) was from Ibidi (Bayern, Germany). Sphingosine-1-phosphate (S-1-P) (#860492) was from Avanti Polar Lipids (Alabaster, AL, USA). VEGF Endothelial Medium Complete Kit (#LL-0003), rhVEGF Life Factor (#LS-1016), and rhFGF basic Life Factor (#LS-1002) were obtained from LifeLine Cell Technology (Frederick, MD, USA). Antigen recovery solution (100×) (#H-330) was from Vector Labs. Cy3 AffiniPure Donkey Anti Rabbit (#711-165-152) was from Jackson Immunoresearch Laboratories (West Grove, PA, USA). ZINC40099027 (Zn27) was purchased from Enamine (Monmouth Jct., NJ, USA). (2-(Dimethylamino)-2-(pyridin-4-yl)ethyl)-3-(2-morpholino-5-(trifluoromethyl)phenyl)urea hydrochloride (M64HCl) was synthesized at the Institute for Therapeutics Discovery and Development, College of Pharmacy, University of Minnesota (Minneapolis, MN, USA). Its molecular formula is C_21_H_27_ClF_35_O_2_, with a molecular weight of 473.9 g/mol. The compound’s structure, synthesis, and quality control methods have been detailed previously [26].

### 2.2. Effects of a FAK Activator on Ulcer Healing

An acetic acid–induced ulcer model was used instead of an indomethacin-induced model to allow precise localization of larger lesions for the investigation of ulcer healing and angiogenesis. Animal procedures were approved by the University of North Dakota Institutional Animal Care and Use Committee under protocol number 2004-3 (Approval Date: 28 April 2020). All mouse experiments were performed with 8–12 weeks old C57Bl/6J male and female mice bred in-house at the University of North Dakota. Mice were bred and housed in temperature-controlled rooms with a 12:12 h light-dark cycle at 23 ± 0.5 °C. Mice had ad libitum access to standard rodent chow and tap water throughout the study. Ischemic ulcers were induced as previously described [27]. Briefly, 8–10-week-old C57BL/6 mice were anesthetized with isoflurane and a laparotomy was performed to expose the jejunum. Ischemic jejunal ulcers were created by placing a 75% acetic acid-saturated circular filter disk (3.14 mm^2^) directly on the antimesenteric serosa for 15 s, without opening the bowel. ZINC40099027 (Zn27), the first FAK activator we identified, is not water-soluble but can be dissolved in dimethyl sulfoxide (DMSO) [27]. To investigate the effects of ZN27 on angiogenesis, male and female mice were randomly assigned to following groups (*n* = 3): untreated mice at day 1 (Day 1 NT) and day 15 (Day 15 NT), as well as DMSO- and ZN27-treated mice at days 3, 5, and 15 received intraperitoneal injections of either ZINC40099027 (900 µg/kg) or a vehicle (DMSO) every six hours for 2, 4, or 14 days, following an initial 24 h period. The dosage and administration schedule for ZN27 were based on our previous study [27]. Body weight was measured daily over the treatment periods. Some animals were sacrificed by cervical dislocation after deep isoflurane anesthesia at day 1 for baseline angiogenesis evaluation. At days 3, 5, and 15, mice were anesthetized by isoflurane anesthesia, blood was drawn by cardiac puncture for assay of serum levels of creatinine and alanine aminotransferase (ALT), and animals were sacrificed by cervical dislocation before removing the jejunal ulcer segment, kidney, and liver for histological examination. The small intestine was opened along the mesenteric attachment and gently washed with phosphate-buffered saline. Mucosal erosions were imaged with an OLYMPUS Q Color 5 digital camera to demonstrate a gross pathology of intestinal lesions. Mouse serum creatinine levels were determined using a Mouse Creatinine kit (Crystal Chem, Elk Grove Village, IL, USA). Serum Alanine Aminotransferase (ALT) levels were assessed using a Mouse ALT ELISA kit (MyBioSource, San Diego, CA, USA).

### 2.3. Histology

Hematoxylin and Eosin (H&E) staining was performed as previously described [25]. Briefly, mouse small intestine, kidney, and liver tissues were fixed in 10% neutralized buffered formalin solution for 48 h, and then transferred into phosphate-buffered saline at 4 °C for storage until processing as paraffin-embedded tissue blocks. Five-micron sections were stained with hematoxylin and eosin. Histologic slides were blindly reviewed by two independent specialists. Histological evaluation included the following parameters: for the kidney—tubular necrosis, inflammatory cell infiltration, and glomerular alterations; and for the liver—vacuolar degeneration, inflammatory cell infiltration, hepatitis, cholestasis, and fibrosis.

### 2.4. Immunofluorescence Staining

Small bowel tissue sections were prepared for immunolabeling as previously described [37]. Briefly, paraffin-embedded tissue sections were deparaffinized, rehydrated, and subjected to antigen recovery by boiling in citrate-based solution (pH 6.0) to expose target proteins. Sections were blocked with 3% donkey serum, 1% BSA, 0.1% Triton X-100, 1X Phosphate-buffered saline (PBS, with Ca^+2^/Mg^+2^) in a moist chamber incubated at RT for 45 min. Tissue samples were then incubated with primary antibody unconjugated anti-phospho-histone H3 (PHH3, 1:200, Sigma-Aldrich) for 90 min at room temperature. After washing the primary antibody, tissues were incubated with fluorochrome-coupled secondary antibody (Cy3 donkey anti rabbit, 1:200, Jackson Immunoresearch Laboratories) and conjugated anti-actin α-smooth muscle-FITC antibody (SMA, 1:500, Sigma-Aldrich) for an hour. Nuclei were labeled with DAPI (Thermo Fisher Scientific). Images were taken at 40× magnification (1 µm interval z-stacks) with Leica DMi8 Stellaris for 6 predetermined areas (2 areas from ulcer bed, 2 areas from ulcer edge, and 2 areas from adjacent tissue). Fluorescence intensity of SMA and the number of PHH3-immunopositive (PHH3+) cells were quantified using Fiji image analysis software (Version 2.14.0, Madison, WI, USA). For quantitative analyses, SMA-positive structures were considered vascular tissue and were restricted to vessels in the mucosa and submucosa; the muscularis externa was excluded to avoid confounding by the smooth muscle layer. PHH3-positive nuclei were counted separately within SMA-positive (vascular) and SMA-negative (non-vascular) regions. The SMA dataset was normalized by subtracting the mean fluorescence intensity of the secondary antibody-only control.

### 2.5. Cell Culture

Primary human umbilical vein endothelial cells (HUVECs; Cat. No. FC0003) were obtained from Lifeline Cell Technology and generously provided by Dr. Grove at the University of North Dakota. HUVECs were cultured using a VEGF Endothelial Medium Complete Kit (Lifeline Cell Technology #LL-0003). This complete kit is composed of 2% serum, basic FGF, VEGF, EGF at a concentration of 5 ng/mL, IGF (15 ng/mL), L-Glutamine (10 mM), hydrocortisone hemisuccinate (1 μg/mL), ascorbic acid (50 µg/mL) and heparin sulfate (0.75 U/mL). HUVECs were sub-cultured at 1:4 cell densities upon reaching confluence using 0.25% trypsin and maintained at 37 °C with 5% CO_2_.

### 2.6. Western Blotting

To investigate the effects of ZN27 on FAK activation and downstream signaling pathways in HUVECs, cells were sparsely seeded (3000 cells/cm^2^) on type I collagen–precoated 150 mm bacteriologic plastic dishes using ELISA coating buffer, allowing the formation of migrating cell islands. When HUVECs reached 50–60% confluence, they were incubated at 37 °C in 5% CO_2_ for 1 h with either 10 nM ZN27 or DMSO (vehicle control), in the absence or presence of the MEK1/2 inhibitor U0126 (10 µM). HUVECs were lysed with 80 uL of protein lysis buffer (50 mM Tris, 150 mM NaCl, 1 mM EDTA, 1 mM EGTA, 1% Triton-X-100, 1% deoxycholic acid, 0.1% SDS, 10% glycerol, and protease and phosphatase inhibitors) to extract protein. Protein concentration was estimated by bicinchoninic acid assay (Thermo Fisher, Waltham, MA, USA). 40 µg of protein lysate were loaded per lane onto 10% SDS-PAGE gels for resolution and transferred onto nitrocellulose membranes as previously described [38]. Membranes were blotted with antibody to Y-397-phosphorylated FAK (1:1000) and T-202/Y-204-phosphorylated ERK1/2 (1:2000). Antibodies to total FAK (1:1000) and total ERK1/2 (1:2000) served as internal references, and densitometry values for pFAK and pERK1/2 were normalized to their respective total proteins. Western blots were performed, and images were detected by the LICOR–Odyssey-Fc imaging system (LI-COR Biosciences, Lincoln, NE, USA). Densitometry was conducted on exposures within the linear range.

### 2.7. HUVECs Sprouting Assay

Three-dimensional collagen matrices were prepared as previously described [39]. Briefly, collagen stocks were made by adding 100% acetic acid to rat tail collagen type-I and aliquoted. Collagen matrices were prepared by adding 10X M199, 5N NaOH, 1X M199, and S-1-P to collagen sequentially. Collagen matrices (80 µL/well) were loaded into a 96-well plate and incubated at 37 °C for 45 min. HUVECs were harvested and resuspended in the necessary volume to adjust the density to 40,000 cell/100 µL with M199 containing VEGF (40 ng/mL) and bFGF (40 ng/mL), ZN27 (10 nM dissolved in a final DMSO concentration of 0.1% *v*/*v*), or DMSO (0.1% *v*/*v*, vehicle control), or no treatment. Then, 100 µL suspension was loaded per well on top of the polymerized collagen matrices. The next day, conditioned media were removed and replaced with 3% glutaraldehyde in PBS for an hour. After one hour fixation, 3% glutaraldehyde was removed, and 0.1% toluidine blue solution was added for 30 min. After staining, toluidine blue was discarded, and the plate was washed in a 1 L beaker filled with distilled water, changing the distilled water every 10 min until the distilled water was clear when removed and stored at 4 °C. HUVECs in collagen matrices, pre-determined 5 areas per well, were imaged at 40× magnification with Leica Thunder Imager System (Leica Microsystems, Deerfield, IL, USA).

### 2.8. Cell Proliferation Assay

Cell proliferation was assayed by determining cell viability using a water-soluble tetrazolium salt (Dojindo Molecular Technology, Rockville, MD, USA). In brief, cells were seeded at 5000 cells per well in 96-well plates in quadruplicates. Cells were allowed to adhere at 37 °C for 24 h. Cells were then treated with ZN27 (10 nM) or DMSO as a vehicle. Cell viability was measured at 0 and 24 h after the treatment. During measurements, 100 µL of fresh medium with 10% CCK8 solution was added per well and incubated at 37 °C for 2 h, according to the manufacturer’s recommendations. Absorbance was measured with a BioTek Epoch spectrophotometer (Winooski, VT, USA) at 450 nm.

### 2.9. In Vitro Monolayer Wound Closure

HUVECs were seeded at 80% confluence into 6-well plates pre-coated with type-I collagen [40]. When the cells reached 100% confluence (48–72 h after seeding), they were wounded with non-barrier autoclaved pipette tips. HUVECs were treated with either ZN27 (10 nM) or a DMSO control for 10 h, either in the absence or presence of hydroxyurea (an anti-proliferative agent, 2 mM) or MEK1/2 inhibitor U0126 (10 µM). Wound images were captured using an inverted light microscope (OLYMPUS CK2, Center Valley, PA, USA) at 0 h and 10 h after wounding. Wound areas were measured with Fiji image analysis software (Version 2.14.0, Madison, WI, USA).

### 2.10. Effects of FAK Activation During Initial NSAIDs Exposure on Subsequent NSAIDs-Induced Reinjury

Due to its limited water solubility, ZN27 is suboptimal for in vivo applications. After conducting the above in vivo and in vitro experiments, we synthesized and characterized a third-generation, water-soluble FAK activator, M64HCl, developed through structure-activity relationship (SAR) studies. M64HCl demonstrates favorable drug-like properties, activates FAK in Caco-2 cells, enhances epithelial sheet migration in vitro, and promotes intestinal mucosal wound healing in rodent models [26,28]. In this experiment, we used M64HCl as a FAK activator. The following animal procedures were approved by the Northeast Ohio Medical University Institutional Animal Care and Use Committee (protocol # 23-04-368; Approval Date: 27 July 2023). Male C57BL/6J mice (24–30 g), aged 10 to 11 weeks (Jackson Laboratory, Bar Harbor, ME, USA), were used in this study. The mice were housed in an animal facility under controlled conditions: temperature maintained at 22 °C, humidity at 50% ± 5%, and a 12 h light/dark cycle with artificial lighting. They had ad libitum access to tap water and commercial chow; diet was identical across treatment groups. Following a one-week acclimatization period, 15 mice were randomly assigned to one of two groups: Re-injury + vehicle or Re-injury + M64HCl. After induction of anesthesia with 1.5–2.5% isoflurane, mice underwent surgery to implant an osmotic pump (model 1007D, ALZET, Cupertino, CA, USA) for continuous infusion of either M64HCl (25 mg/kg/day) or saline (vehicle). Each pump was filled with the designated solution and implanted subcutaneously through a small incision just posterior to the scapulae. A pocket was created using a hemostat, and the pump was inserted with the cap flush inside. The incision was closed with wound clips. Immediately after implantation, all mice received a subcutaneous injection of indomethacin (10 mg/kg), an NSAID, to induce intestinal injury. This approach represents a well-established model of gastrointestinal (GI) injury [41]. The Omnipump, containing either M64HCl or vehicle, was removed seven days later. After an additional seven days (14 days after the initial indomethacin dose), a second subcutaneous injection of indomethacin (10 mg/kg) was administered. One day after this second dose, the animals were euthanized for analysis. The mice were closely monitored for signs of severe illness, including altered activity levels, vocalization, ruffled fur, rectal bleeding, hunched posture, and refusal to eat or drink. If any of these signs occurred, the animals were euthanized. Since the Omnipump was removed seven days post-implantation, no residual drug was present at the time of the second indomethacin administration. This design enables assessment of whether prior M64HCl treatment reduces intestinal injury caused by a second indomethacin challenge after the initial injury has resolved. Euthanasia was conducted under deep anesthesia using 3–4% isoflurane, followed by cervical dislocation. The entire small intestine was quickly excised, and all ulcers were photographed. The area of each ulcer was measured using ImageJ software (Version 1.54q, National Institutes of Health, Bethesda, MD, USA), and the total ulcer area per mouse was calculated.

### 2.11. Statistical Analysis

Statistical analysis was performed using GraphPad Prism 9 (GraphPad Software, La Jolla, CA, USA). Data are presented as mean ± standard deviation (SD). For the experiment described in Section 2.2, total of three mice (male and female) were assigned to each group (untreated mice at day 1 (Day 1-NT) and day 15 (Day 15-NT), DMSO- and ZN27-treated mice at day 3 (Day 3-DMSO and Day 3-ZN27), day 5 (Day 5-DMSO and Day 5-ZN27), and day 15 (Day 15-DMSO and Day 15-ZN27) and there were no exclusions. For the experiment described in Section 2.10, 15 mice were initially assigned to either the Re-injury + Vehicle group or the Re-injury + M64HCl group. Four mice in each group died after receiving the first dose of indomethacin (10 mg/kg). In addition, two mice in the Re-injury + M64HCl group fought with each other, resulting in severe back skin injuries; therefore, they were excluded from further experiments. Consequently, data from 11 mice in the Re-injury + Vehicle group and 9 mice in the Re-injury + M64HCl group were analyzed. Comparisons between two groups were made using an unpaired two-tailed t-test. For comparisons involving three or more groups, a one-way ANOVA was first conducted. If the ANOVA indicated a significant difference, Tukey’s multiple comparison test was used for post hoc pairwise comparisons. A *p*-value of less than 0.05 was considered statistically significant.

## 3. Results

### 3.1. FAK Activation Promoted Intestinal Mucosal Healing and Enhanced Both Blood Vessel Formation and Cell Proliferation in the Ulcer Bed and Ulcer Edge After 2 Days (Day 3) and 4 Days (Day 5) of Treatment in a Murine Model of Jejunal Ischemic Ulcers

**Figure 1:**
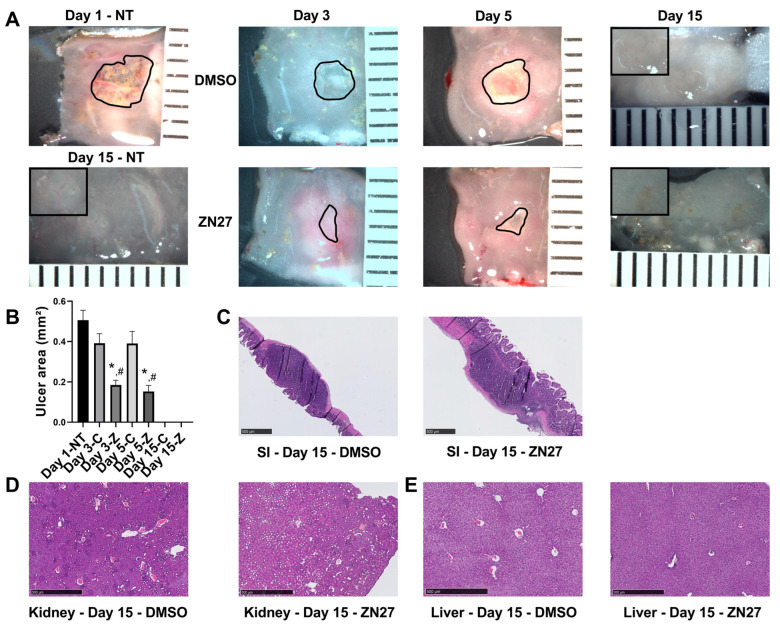
ZN27 promotes intestinal mucosal healing in a murine model of acetic acid-induced ulceration. (**A**) Representative images of jejunal ischemic ulcers in untreated mice at day 1 (Day 1 NT) and day 15 (Day 15 NT), as well as in DMSO- and ZN27-treated mice at days 3, 5, and 15. Black outlines indicate the ulcer circumference. Inset: higher-magnification image of an ischemic ulcer at day 15. Ulcer areas were not measured at day 15 due to the absence of open wounds. (**B**) Quantification of total small intestinal ulcer areas in mice treated with DMSO or ZN27 at days 3 and 5, compared with ulcer area in mice sacrificed at day 1 prior to treatment. ZN27 significantly reduced ulcer size after 2 and 4 days of intraperitoneal injections every 6 h (*n* = 3, * *p* < 0.01 vs. Day 1, # *p* < 0.05 vs. DMSO). (**C**) Representative hematoxylin and eosin (H&E) staining of jejunal ischemic ulcers in mice treated with DMSO or ZN27 at day 15. (**D**) Representative H&E images of kidney tissue from DMSO- and ZN27-treated mice at day 15. (**E**) Representative H&E images of liver tissue from DMSO- and ZN27-treated mice at day 15. No notable differences were observed in kidney or liver structure between treatment groups following fourteen days of DMSO or ZN27 administration, beginning one day after ischemic ulcer induction via topical serosal acetic acid (scale bars, 500 µm). Abbreviations: NT, no treatment; C, vehicle control-DMSO; Z, ZN27.

Application of acetic acid to the serosa induced well-demarcated ulcer craters in the intestinal lumen. Figure 1A shows representative images of acetic acid-induced intestinal ulcers in untreated, vehicle-treated, and FAK activator ZN27-treated mice. Ulcer areas were quantified from digital images using ImageJ by manually tracing their contours. Consistent with our previous findings [27], quantification (Figure 1B) showed the largest ulcer area at day 1 after injury induction. In mice treated intraperitoneally with ZN27 every 6 h for 2 or 4 days, ulcer areas were significantly smaller than in DMSO (vehicle)-treated controls or untreated mice, indicating that FAK activation by ZN27 promotes ischemic ulcer healing. These findings are consistent with previous observations [25,26,27,28,29]. By day 15, histological analysis showed complete healing of ulcers in all groups (Figure 1C).

Histological examination of the kidney (Figure 1D) and liver (Figure 1E) tissues revealed no structural alterations between the DMSO-treated and ZN27-treated groups. Serum creatinine levels were within the normal range (0.06–1.6 mg/dL, Crystal Chem) across all groups, with no significant differences (Table 1). Although DMSO-treated mice showed slightly higher ALT levels compared to untreated controls (19.19 ± 1.37 U/L vs. 8.80 ± 0.70 U/L), ALT remained within the physiological range (7.63–53.1 U/L) for all groups (Table 1).

**Figure 2:**
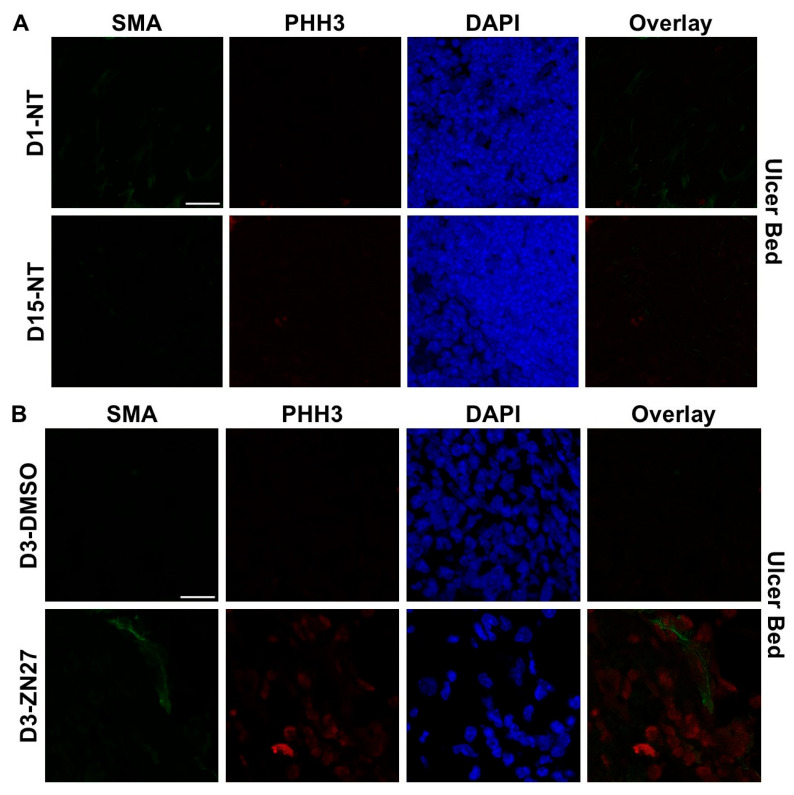

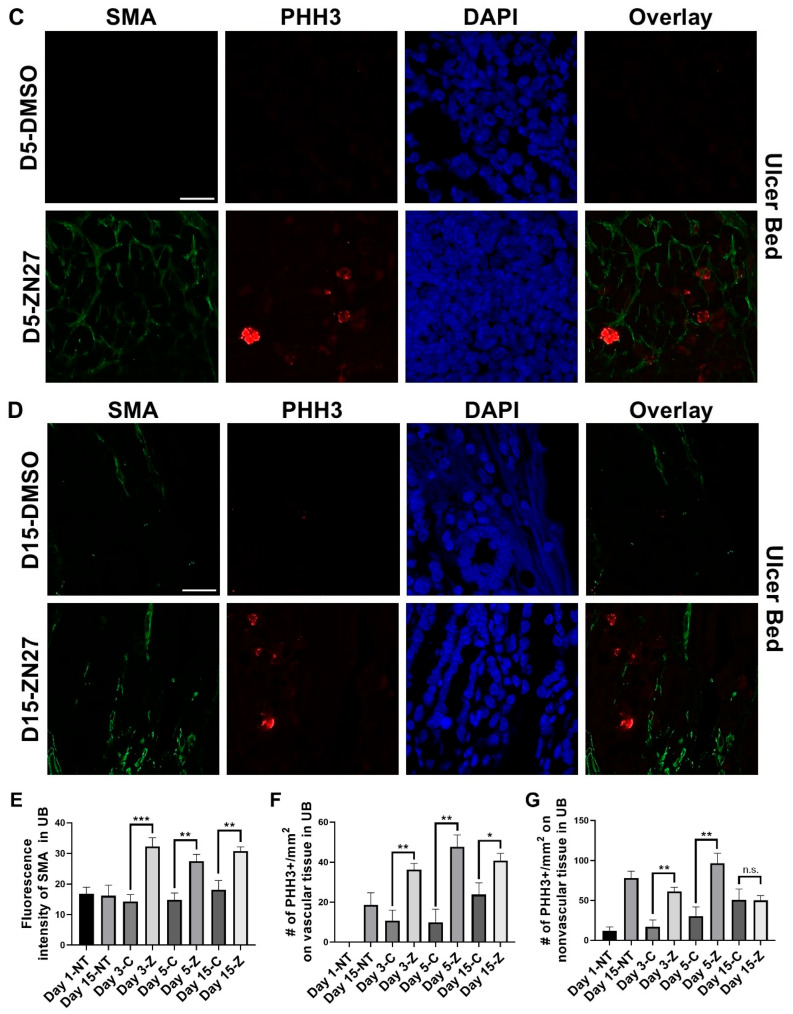
ZN27 enhanced blood vessel formation and proliferation of both vascular and nonvascular tissues in the ulcer bed on days 3, 5, and 15, but did not affect proliferation of nonvascular tissues on day 15 in a murine ischemic ulcer model. (**A**) Representative images of murine ischemic jejunal injuries without treatment at day 1 and day 15. (**B**) Representative images of DMSO-treated and ZN27-treated murine ischemic jejunal injuries at day 3. (**C**) Representative images of DMSO-treated and ZN27-treated murine ischemic jejunal injuries at day 5. (**D**) Representative images of DMSO-treated and ZN27-treated murine ischemic jejunal injuries at day 15. (**E**) Quantification of mean fluorescence intensity of α-SMA in the ulcer bed at days 1, 3, 5, and 15 (*n* = 3, ** *p* < 0.01, *** *p* < 0.001). (**F**) Quantification of PHH3-positive cells per mm^2^ in vascular tissue at days 1, 3, 5, and 15 (*n* = 3, * *p* < 0.05, ** *p* < 0.01). (**G**) Quantification of PHH3-positive cells per mm^2^ in nonvascular tissue at days 1, 3, 5, and 15 (*n* = 3, ** *p* < 0.01, n.s.: not significant). Abbreviations: SMA, alpha-smooth muscle actin; PHH3, phosphorylated histone H3; UB, ulcer bed; NT, no treatment; C, DMSO vehicle control; Z, ZN27; scale bars, 200 µm.

**Figure 3:**
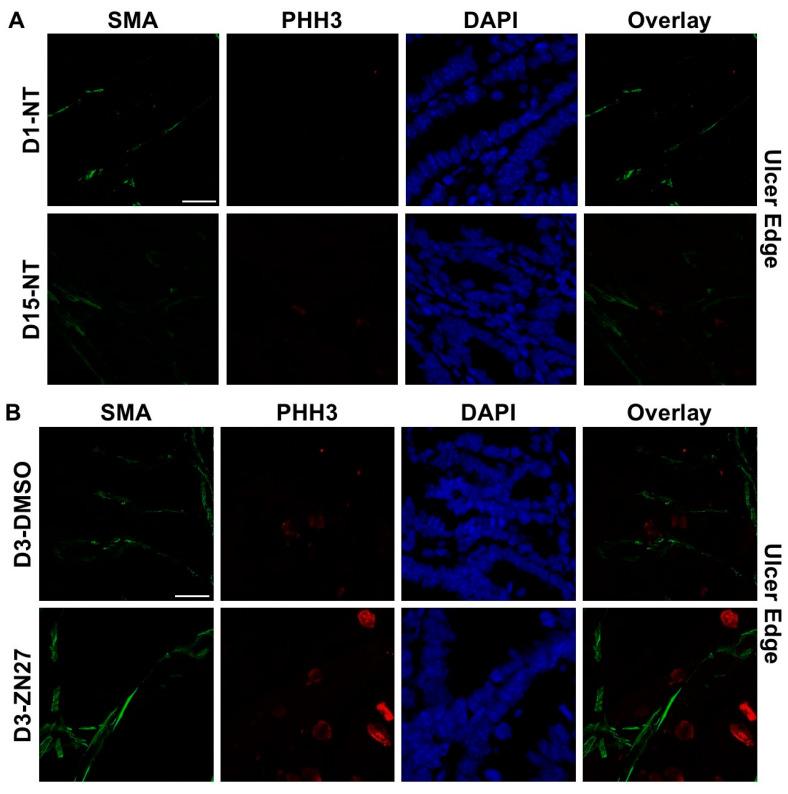

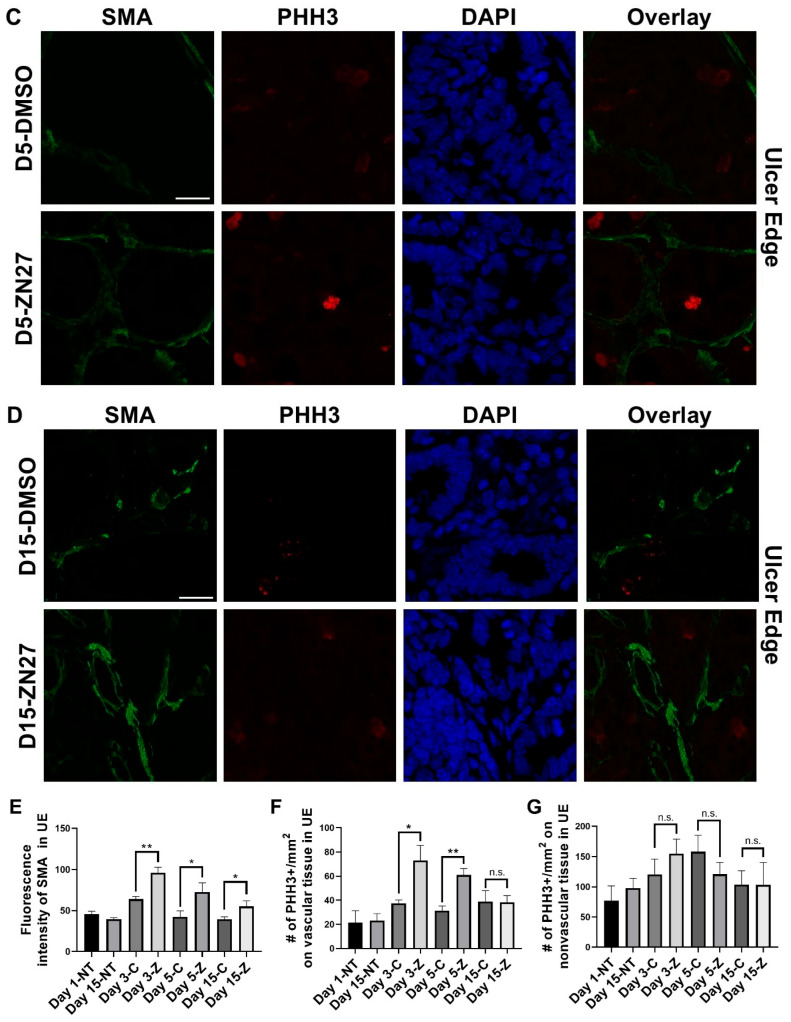
ZN27 promoted blood vessel formation at the ulcer edge on days 3, 5, and 15. It enhanced vascular tissue proliferation at the ulcer edge on days 3 and 5, but not on day 15, and showed no effect on non-vascular tissue proliferation at any treatment time point in a murine ischemic ulcer model. (**A**) Representative images of the murine ischemic ulcer edge without treatment on days 1 and 15. (**B**) Representative images of the ulcer edge in DMSO-treated and ZN27-treated mice on day 3. (**C**) Representative images of the ulcer edge in DMSO-treated and ZN27-treated mice on day 5. (**D**) Representative images of the ulcer edge in DMSO-treated and ZN27-treated mice on day 15. (**E**) Quantification of mean fluorescence intensity of α-SMA in the ulcer edge on days 1, 3, 5, and 15 (*n* = 3, * *p* < 0.05, ** *p* < 0.01). (**F**) Quantification of PHH3-positive cells per mm^2^ in vascular tissue at the ulcer edge on days 1, 3, 5, and 15 (*n* = 3, * *p* < 0.05, ** *p* < 0.01, n.s.: not significant). (**G**) Quantification of PHH3-positive cells per mm^2^ in avascular tissue at the ulcer edge on days 1, 3, 5, and 15 (*n* = 3, n.s.: not significant). Abbreviations: SMA, alpha-smooth muscle actin; PHH3, phosphorylated histone H3; UE, ulcer edge; NT, no treatment; C, DMSO vehicle control; Z, ZN27; scale bars, 200 µm.

To investigate whether FAK activation promotes ulcer healing through angiogenesis and proliferation, we performed immunostaining. α-Smooth muscle actin (α-SMA), a contractile filamentous protein and vascular smooth muscle marker, was used to identify blood vessels [42], while PHH3 served as a mitotic proliferation marker [43]. Representative immunofluorescence images of ulcer bed (Figure 2) and ulcer edge (Figure 3) areas were obtained in untreated mice at days 1 and 15, and in DMSO- or ZN27-treated mice at days 3, 5, and 15. Quantification of mean α-SMA fluorescence intensity showed increased expression in ZN27-treated mice compared to DMSO-treated controls at days 3, 5, and 15 in both ulcer bed and edge regions (Figure 2E and Figure 3E). The number of PHH3-positive cells was higher in ZN27-treated mice compared to controls at days 3, 5, and 15 in the ulcer bed, and at days 3 and 5 in the ulcer edge (Figure 2F and Figure 3F). No significant changes in PHH3-positive cells were observed at day 15 in the ulcer bed or at days 3, 5, and 15 in the ulcer edge (Figure 2G and Figure 3G). These data suggest that FAK activation with ZN27 increased blood vessel density at days 3, 5, and 15 in the ulcer bed, and at days 3 and 5 in the ulcer edge, accompanied by enhanced vascular smooth muscle cell proliferation but not enhanced epithelial proliferation.

### 3.2. ZN27 Activates FAK and Its Downstream Effector—ERK1/2 in HUVECs

**Figure 4:**
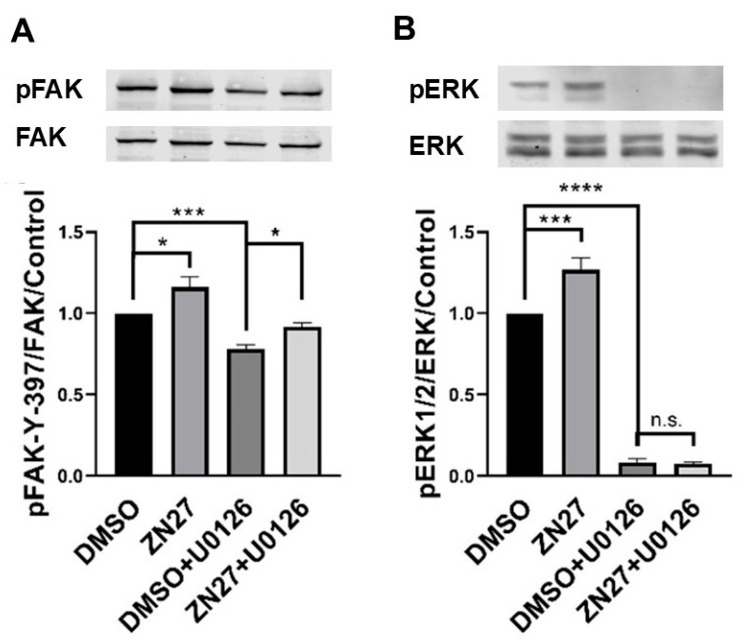
The MEK inhibitor U0126 prevented ZN27-induced phosphorylation of ERK1/2 but did not alter ZN27-stimulated phosphorylation of FAK-Y397 in HUVECs. Cells were treated with either 0.1% DMSO (vehicle control) or 10 nM ZN27 for one hour, in the presence or absence of U0126 (10 µM). Total protein levels were used as a loading control for Western blot analyses. (**A**) Representative blots and quantification of pFAK-Y397/FAK fold change in HUVECs. (**B**) Representative blots and quantification of pERK1/2/ERK1/2-fold change in HUVECs. *n* = 7, * *p* < 0.05, *** *p* < 0.001, **** *p* < 0.0001, n.s.: not significant.

Our previous studies showed that ZN27 increases phosphorylation of both FAK and its downstream substrate ERK1/2 in Caco-2 cells [44]. Consistently, in HUVECs, ZN27 (10 nM) increased phosphorylation of FAK-Y397 by 16.1 ± 6.2% (*p* < 0.05; Figure 4A) and ERK1/2-T202/Y204 by 26.8 ± 7.0% (*p* < 0.001; Figure 4B). The MEK1/2 inhibitor U0126 (10 µM) reduced phosphorylation of ERK1/2-T202/Y204 by 91.5 ± 1.9% (*n* = 7, *p* < 0.05). Notably, ZN27 treatment in the presence of U0126 still increased FAK-Y397 phosphorylation by 17.1 ± 4.3% (*n* = 7, *p* < 0.05; Figure 4A) but failed to enhance ERK1/2 phosphorylation (Figure 4B). These results demonstrate that ZN27 activates FAK in HUVECs and that ERK1/2 activation occurs downstream of FAK.

### 3.3. ZN27 Increased the Number of Sprouts in HUVECs

**Figure 5:**
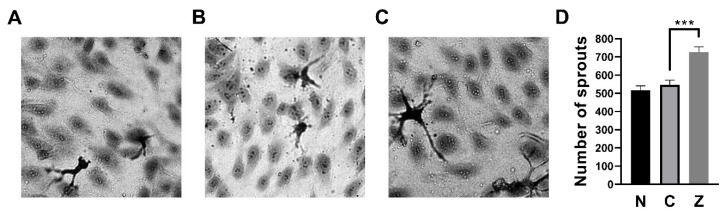
ZN27 increased sprouting in HUVECs. (**A**–**C**) Representative wound images of HUVECs under the following conditions: no treatment, DMSO (vehicle control), and ZN27 (10 nM). All images were captured at 40× magnification. (**D**) Treatment with ZN27 (10 nM) significantly increased the number of sprouts in HUVECs compared with DMSO-treated cells (*n* = 6, pooled from 3 independent experiments with similar results; *** *p* < 0.001). N, no treatment, C, vehicle control (DMSO), Z, ZN27.

The endothelial sprouting assay was performed to investigate whether ZN27 has pro-angiogenic effects. Representative images of HUVEC sprouting assays are shown in Figure 5A–C. ZN27 (10 nM) increased the number of sprouts by 32.6 ± 8.2% compared to DMSO-treated controls (*n* = 6, *p* < 0.001; Figure 5D), suggesting that ZN27 promotes pro-angiogenic sprouting behavior in HUVECs.

### 3.4. FAK Activation Did Not Alter Proliferation but Stimulated Migration Through ERK1/2 Activation in HUVECs

**Figure 6:**
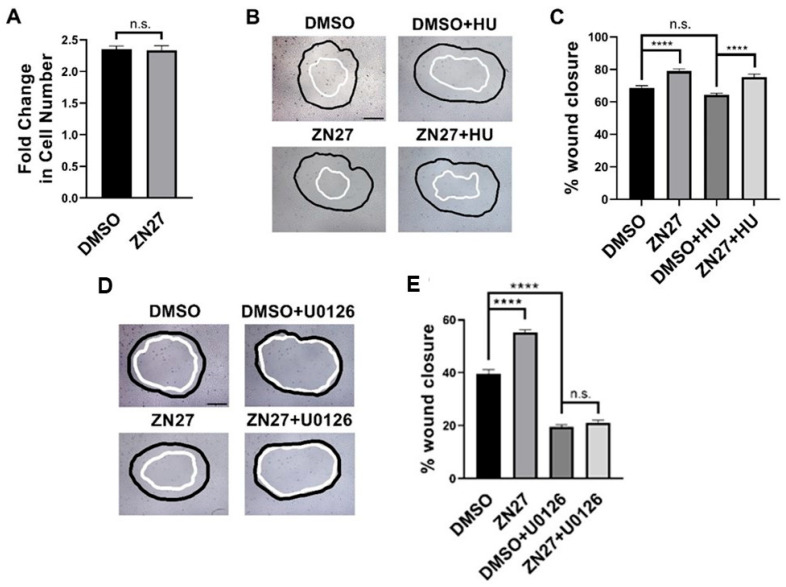
FAK activation did not alter proliferation but stimulated wound closure through ERK1/2 activation in HUVECs. (**A**) The number of cells in the ZN27 group did not change in comparison to the DMSO control group (*n* = 44, *p* > 0.05, n.s.: not significant). (**B**) Typical wound images for HUVECs, treated with DMSO, ZN27 (10 nM), DMSO + Hydroxyurea (HU, 2 mM), or ZN27 (10 nM) + HU (2 mM). (**C**) ZN27 at 10 nM accelerates circular wound closure in HUVEC monolayers on collagen I even when proliferation is blocked by 2 mM hydroxyurea (*n* = 10–11, pooled from 3 separate studies with similar results, **** *p* < 0.0001, n.s.: not significant). (**D**) Typical wound images for HUVECs treated with DMSO, ZN27, DMSO + U0126, and ZN27 + U0126. (**E**) ZN27 at 10 nM accelerates circular wound closure, however U0126 treatment (10µM) blocked wound closure induced by ZN27 in HUVEC endothelial cell monolayers on collagen I. (n = 10–11, pooled from 3 separate studies with similar results, **** *p* < 0.0001, n.s.: not significant).

We next examined whether FAK activation enhances proliferation, migration, or both in HUVECs. Proliferation assays showed no significant difference in cell number between ZN27- and DMSO (vehicle)-treated cells after 24 h (2.35 ± 0.05 vs. 2.33 ± 0.07, *n* = 44, *p* = 0.85; Figure 6A), indicating that ZN27 does not promote HUVEC proliferation.

Wound images were acquired at 0 and 10 h. The black outlines indicate the wound boundaries at 0 h, while the white outlines represent the wound boundaries after 10 h. All images were captured at 10× magnification, with scale bars representing 200 µm.

Migration was assessed using a wound healing assay (Figure 6B), with or without hydroxyurea (2 mM) to block proliferation [45]. The presence of 2 mM hydroxyurea did not reduce overall wound closure in HUVECs. ZN27 increased wound closure by 15.3 ± 3.1% compared to DMSO controls (*n* = 23, *p* < 0.0001; Figure 6C). In the presence of hydroxyurea, ZN27 still enhanced wound closure by 17.1 ± 3.2% (*n* = 24, *p* < 0.0001; Figure 6C). These findings indicate that ZN27 stimulates HUVEC migration.

The MEK1/2 inhibitor U0126 treatment alone reduced wound closure by 50.3 ± 2.4% (*n* = 11, *p* < 0.0001, Figure 6D,E). In addition, U0126 abolished the stimulatory effect of ZN27 on wound closure (*n* = 11; Figure 6D,E). These results indicate that ZN27 promotes HUVEC migration through ERK1/2 activation.

### 3.5. Mice Recovered from M64HCl-Treated, Indomethacin-Induced Intestinal Injury Showed Resistance to Subsequent Indomethacin Challenge

**Figure 7:**
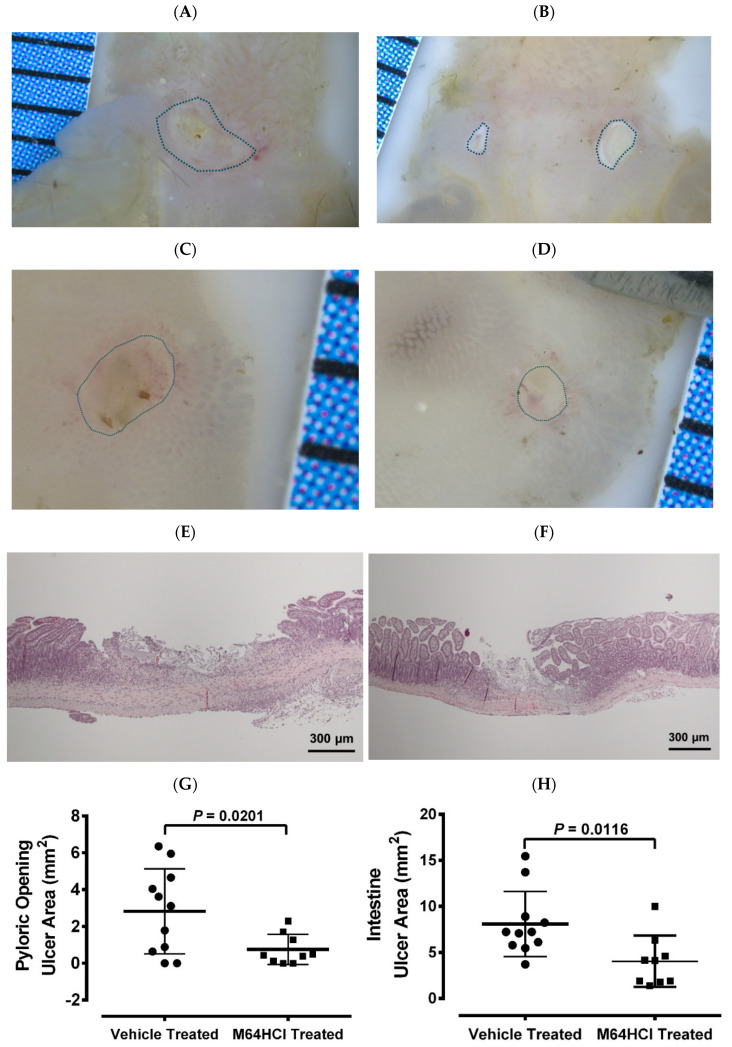
The M64HCl-healed mucosa exhibits resistance to re-injury. Mice that had previously recovered from an indomethacin-induced injury—treated either with vehicle (saline) or with concomitant M64HCl for a week—were re-administered indomethacin two weeks later. The animals were euthanized one day after the re-administration, without additional drug treatment. Panels (**A**,**B**) show representative images of ulcers in the pyloric opening from vehicle-treated and M64HCl-treated re-injury groups, respectively. Panels (**C**,**D**) present representative images of ulcers in the small intestine from vehicle-treated and M64HCl-treated re-injury groups, respectively. Panels (**E**,**F**) show representative hematoxylin and eosin (H&E) staining of ulcers in the small intestine from the vehicle-treated and M64HCl-treated re-injury groups, respectively. Quantification of the total ulcerated area in the pyloric opening (**G**) and the small intestine (**H**) revealed a significant reduction in mice whose initial injuries had been treated with M64HCl (*n* = 9) compared with those treated with vehicles (*n* = 11). Each division on the ruler represents one millimeter.

In a mouse model of indomethacin-induced small intestinal injury, animals were treated with either the next-generation FAK activator M64HCl or vehicle. After recovery from the initial injury, both groups were subjected to a secondary indomethacin challenge. Representative images of ulcers in the pyloric opening (Figure 7A,B) and small intestine (Figure 7C,D) are shown. Figure 7E,F showed representative hematoxylin and eosin staining of ulcers in the small intestine from the vehicle-treated and M64HCl-treated re-injury groups, respectively. Quantification demonstrated that mice pretreated with M64HCl during the initial injury exhibited significantly smaller total ulcer areas in both the pyloric opening (Figure 7G) and small intestine (Figure 7H) compared to vehicle-treated controls, despite no further M64HCl administration. These findings suggest that FAK activation not only accelerates mucosal healing but also confers resistance to subsequent NSAID-induced injury.

## 4. Discussion

NSAIDs are widely prescribed due to their analgesic and anti-inflammatory effects. They are effective in managing fever, as well as pain and inflammation associated with rheumatoid arthritis, gout, and osteoarthritis. However, chronic NSAID use is strongly linked to gastrointestinal (GI) complications. Graham et al. [46] reported that over 70% of long-term NSAID users develop intestinal inflammation, bleeding, or protein loss. Similarly, another study found that 75% of healthy volunteers developed macroscopic small intestinal injury within two weeks of slow-release diclofenac administration, with 40% showing mucosal breaks [47]. Such injuries can lead to occult GI bleeding, and NSAIDs are implicated in 10–15% of cases of iron-deficiency anemia [1]. Persistent impairment of the mucosal barrier has been associated with poor clinical outcomes, diminished quality of life, and elevated risks of surgical complications and mortality [48].

Successful GI mucosal healing requires coordinated epithelial migration, proliferation, and angiogenesis [49,50]. Focal adhesion kinase (FAK), a cytoplasmic tyrosine kinase, plays a central role in integrin and growth factor signaling, regulating epithelial cell migration and angiogenesis [51,52]. Interestingly, FAK activity is often reduced at the leading edge of epithelial wounds [53], suggesting that targeted FAK activation may accelerate repair. Although prior studies support FAK’s involvement in re-epithelialization and vascular remodeling, most interventions have relied on indirect or non-specific modulation. Thus, the therapeutic impact of selective FAK activation on mucosal healing and resistance to subsequent reinjury has remained largely unexplored. To address this, our group developed a series of FAK activators—initially ZN27 [27] and, more recently, M64HCl [26,28], and investigated their therapeutic potential and underlying mechanisms in gastrointestinal (GI) injury.

Consistent with our earlier findings [25,26,27,28,29], the present study demonstrated that ZN27 treatment significantly accelerated ischemic ulcer healing within 2–4 days. This effect appears to result primarily from enhanced epithelial cell migration. The current work further extends previous studies in this model to longer duration. By day 15, ulcers in all treatment groups had completely healed. Importantly, ZN27 exhibited a favorable safety profile: histological examination revealed no structural abnormalities in renal or hepatic tissues, and serum creatinine and ALT levels remained within normal ranges. Immunofluorescence staining further showed that ZN27 treatment increased blood vessel density in the ulcer bed (days 3, 5, 15) and ulcer margins (days 3, 5), along with enhanced proliferation. Subsequent in vitro experiments confirmed that ZN27 activates FAK and downstream ERK1/2 signaling in HUVECs. Endothelial sprouting assays revealed increased sprout formation, confirming pro-angiogenic activity, while wound-healing assays showed enhanced HUVEC migration via ERK1/2 activation. Interestingly, proliferation was not affected. Collectively, these findings indicate that ZN27 promotes wound healing at least in part through pro-angiogenic effects, in addition to facilitating epithelial migration, as shown in our previous studies [25,26,27,28,29].

Re-epithelialization and neovascularization are central to GI ulcer healing. Re-epithelialization establishes the essential barrier that separates the body from harmful luminal contents. Angiogenesis facilitates the delivery of oxygen and nutrients, and expedites waste clearance, while granulation tissue offers a scaffold for cell migration and extracellular matrix deposition [30]. Conversely, the changing matrix can affect further angiogenic [54] or epithelial migratory [55] behavior. Substances that promote angiogenesis often also facilitate wound healing [30]. For example, local injection of basic fibroblast growth factor (bFGF) in rats with lip mucosal wounds enhanced angiogenesis, granulation tissue formation, and healing [56].

FAK is a key mediator of integrin and growth factor signaling, with several studies confirming its critical role in angiogenesis. Sun et al. utilized FAK knockout mice and FAK-silenced MS1 cells to demonstrate that FAK upregulates VEGFR2 expression, thereby promoting angiogenesis [32]. Endothelial-specific FAK overexpression promoted angiogenesis in transgenic mice [33], whereas endothelial-specific FAK deletion resulted in defective angiogenesis and embryonic lethality [34].

ZN27 activates FAK and stimulates ERK1/2 signaling. FAK leads to activation of ERK via the Grb2-Sos-Ras pathway. FAK interacts with key signaling proteins like Src and Grb2, which then recruit Sos. This recruitment activates the Ras GTPase, which in turn activates Raf kinase, leading to the phosphorylation of MEK and subsequently ERK [57,58]. The ERK pathway regulates cell survival and migration, and its inhibition can impair wound healing. For example, MEK inhibition reduces angiogenesis in HUVECs [59], suppresses keratinocyte migration [60], and delays skin wound healing [61]. Similarly, ERK inhibition blocks HUVEC migration under hypoxia [62] or lysophosphatidylglycerol stimulation [63], in part by disrupting Rac1-mediated actin remodeling.

While our data confirmed that ZN27 enhances HUVEC migration but not proliferation, the role of FAK in proliferation is complex and context-dependent. FAK can promote proliferation by activating YAP nuclear translocation or inhibiting JNK signaling [64], but in settings of inadequate adhesion, FAK may suppress proliferation by promoting cytoskeletal tension and focal adhesion formation [65]. Thus, FAK may exert dual, context-specific effects on proliferation. Taken together with our previous studies in human Caco-2 epithelial cells [27], the PHH3 staining in the present study, along with prior Ki67 staining in tissues [27], suggests that FAK activation may promote vascular smooth muscle cell proliferation in this context, but does not appear to stimulate epithelial or endothelial proliferation.

These results suggest that FAK activation not only heals mucosal injury more rapidly but also more effectively, resulting in higher quality healed mucosa that more effectively resists recurrent NSAID-induced intestinal injury. Treatment with M64HCl during the initial indomethacin challenge reduced susceptibility to reinjury after re-exposure. Importantly, M64HCl has a relatively short half-life [26] and was not administered during the week before or after the second dose challenge. The quality of ulcer healing strongly influences recurrence risk: well-healed ulcers are more resistant to reinjury, whereas poor-quality healing predisposes to relapse and complications [15,16,17,18]. In our ischemic ulcer model, FAK activation accelerated healing and enhanced angiogenesis at both ulcer margins and beds. These vascular changes likely strengthened tissue resistance to reinjury. Conversely, delayed healing impairs barrier function, promotes bacterial translocation, and leads to chronic inflammation, fibrosis, and structural weakness [66]. Therefore, by promoting epithelial restitution and angiogenesis, FAK activation accelerates healing, prevents poor vascularization and scar formation, and reduces the risk of recurrent NSAID-induced injury. Mechanistically, these findings support a model in which FAK activation coordinates epithelial restitution and vascular remodeling through ERK1/2-dependent migration and sprouting. From a translational standpoint, FAK activators such as ZN27 and M64HCl merit further evaluation as candidate therapeutics to enhance mucosal repair and reduce recurrence risk in patients who require long-term NSAID therapy.

Although ZN27 and M64HCl demonstrate robust activity in rodent models, both compounds remain at an early preclinical stage. Translation toward clinical testing will require systematic pharmacokinetic and safety evaluation in large animals, assessment of off-target effects, and optimization of dosing regimens and formulations suitable for chronic use. Because ZN27 has limited aqueous solubility, our work also highlights M64HCl as a more clinically tractable scaffold for future development. If safety and efficacy are confirmed, FAK activators could be tested clinically in high-risk populations such as patients with a prior history of NSAID-associated small intestinal ulcers who must continue NSAID therapy, or individuals with inflammatory conditions requiring long-term NSAID exposure. In such settings, FAK activation might be used adjunctively with existing gastroprotective measures to enhance mucosal healing and reduce recurrence.

Taken together, our three complementary models provide a unified view of how FAK activation promotes gastrointestinal mucosal repair. In the ischemic jejunal ulcer model, pharmacologic FAK activation accelerated early healing and increased vascular density and granulation tissue formation. The HUVEC studies further demonstrated that ZN27 directly activates FAK–ERK1/2 signaling, enhances endothelial migration, and promotes sprouting behavior, providing a mechanistic link between FAK activation and angiogenic remodeling.

This study has several limitations. First, we did not include a FAK inhibitor arm in the in vivo ischemic ulcer model; although prior genetic and pharmacologic loss-of-function studies demonstrate that FAK is essential for epithelial migration and angiogenesis, future work directly comparing FAK activation and inhibition in parallel in vivo will further refine the mechanistic role of FAK signaling. Second, vascular identification in murine tissues was based on α-SMA, which labels mature vessels but does not distinguish endothelial structures with the specificity of CD31 or other endothelial markers; incorporating endothelial markers and direct FAK immunostaining in future studies will help define the cellular specificity of FAK activation during ulcer repair. Third, endothelial sprouting was used as the primary in vitro readout of angiogenic behavior; additional angiogenesis assays—such as tube formation—would provide complementary functional validation and help differentiate sprouting from potential endothelial-to-mesenchymal transition. Despite these limitations, the integrated in vivo and in vitro data strongly support a pro-healing, pro-angiogenic role for FAK activation in gastrointestinal mucosal repair.

## 5. Conclusions

This study demonstrates that FAK activation markedly accelerates ischemic ulcer healing, likely in part through enhanced pro-angiogenic effects. Notably, FAK activation during the early phase of mucosal repair also reduced susceptibility to recurrent NSAID-induced intestinal injury, suggesting it promotes more durable and higher-quality healing. Considering its favorable safety profile and efficacy in rodent models, further evaluation in large animal studies is warranted to establish its therapeutic potential.

## Figures and Tables

**Table 1:** Serum creatinine and ALT levels in mice under different treatment conditions.

Parameters	Normal Range	No Treatment (*n* = 3)	DMSO-Treated (*n* = 3)	ZN27-Treated (*n* = 3)
Serum creatinine	0.06–16 mg/dL	0.11 ± 0.02	0.14 ± 0.06	0.13 ± 0.03
Serum ALT	7.63–53.1 U/L	8.80 ± 0.70	19.19 ± 1.37	17.95 ± 3.31

## Data Availability

Data will be made available upon request.
